# A Low-Complexity Euclidean Orthogonal LDPC Architecture for Low Power Applications

**DOI:** 10.1155/2015/327357

**Published:** 2015-04-29

**Authors:** M. Revathy, R. Saravanan

**Affiliations:** ^1^Department of Electronics and Communication Engineering, PSNA College of Engineering and Technology, Dindigul 624622, India; ^2^Department of Computer Science and Engineering, RVS Educational Trust Group of Institutions, Dindigul 624005, India

## Abstract

Low-density parity-check (LDPC) codes have been implemented in latest digital video broadcasting, broadband wireless access (WiMax), and fourth generation of wireless standards. In this paper, we have proposed a high efficient low-density parity-check code (LDPC) decoder architecture for low power applications. This study also considers the design and analysis of check node and variable node units and Euclidean orthogonal generator in LDPC decoder architecture. The Euclidean orthogonal generator is used to reduce the error rate of the proposed LDPC architecture, which can be incorporated between check and variable node architecture. This proposed decoder design is synthesized on Xilinx 9.2i platform and simulated using Modelsim, which is targeted to 45 nm devices. Synthesis report proves that the proposed architecture greatly reduces the power consumption and hardware utilizations on comparing with different conventional architectures.

## 1. Introduction

Low-density parity-check (LDPC) codes, introduced by Gallager [1] during the 1960s, are a class of linear block LDPC codes. The excellent error correction properties of low-density parity- check (LDPC) codes have gained great significance in the research fields. Among various error correction codes, LDPC codes are chosen as better codes, closer to the Shannon limit. LDPC codes achieve 0.04 dB of Shannon's limit. Compared to other codes, LDPC codes with iterative decoding are easy to implement. These codes have an enhanced error correcting capability. The name “LDPC” comes from the characteristic of their parity-check matrix which contains only a few 1s in comparison to the amount of 0s. Their main advantage is that they provide a performance which is very close to the capacity for a lot of different channels and linear time complex algorithms for decoding.

LDPC codes have certain advantages over turbo codes (the best codes with BER closer to Shannon capacity, so far). The first merit is that the decoding of LDPC codes fully follows parallelism which performs at a higher speed. Secondly, very low-complexity decoders are more enough and suitable for these codes. Thirdly, LDPC decoding is verifiable; that is, decoding to a correct code word can be verified for accuracy.

Moreover, LDPC codes are more suitable for implementation in applications that employ parallelism. Therefore, these codes can be employed in several standards including WiMax (IEEE 802.16) and different high speed applications in which iterative message-passing algorithms are mostly implemented in parallel. LDPC implements parallelism in the decoding process thereby achieving high decoding throughput. LDPC codes consecutively decode both rows and columns and therefore these codes are more suitable for iterative decoding, hence referred to as iterative decoders.

LDPC codes can be represented in two ways. The first method is in the form of matrices, similar to all other linear block codes, and the second way of representation is in the form of graphs. There are several algorithms proposed to decode LDPC codes and each algorithm has been designed autonomously and comes under different names as a matter of fact. Some of the widespread LDPC decoders used commonly are the sum-product algorithm, belief propagation algorithm, and message-passing algorithm.

In this paper, a low-complexity Euclidean orthogonal LDPC architecture has been proposed for low power applications.  Section 2 reviews some of the related LDPC based decoding techniques.  Section 3 explains the proposed LDPC decoder, algorithm, and internal modules design in detail. The results and discussion are placed in Section 4 and Section 5 concludes our work.

## 2. Related Works

Darabiha et al. [2] investigated the VLSI architectures for low-density parity-check (LDPC) decoders amenable to low-voltage and low power operation. They designed a highly parallel decoder architecture with low routing overhead. Secondly, they proposed an efficient method to detect early convergence of the iterative decoder and terminate the computations, thereby reducing dynamic power. A bit-serial fully parallel LDPC decoder fabricated in a 0.13 *μ*m CMOS process was reported and the above techniques were implemented to show the effect of power consumption. With early termination, the prototype decoded 10.4 pJ/bit/iteration, while performing within 3 dB of the Shannon limit at a BER of 10^−5^ and a throughput of 3.3 Gbps. If operated from a 0.6 V supply, the energy consumption can be further reduced to 2.7 pJ/bit/iteration with a total throughput of 648 Mbps, due to the highly parallel architecture.

Mansour and Shanbhag [3] proposed high-throughput memory-efficient decoder architecture for LDPC codes based on turbo decoding algorithm. A new merged-schedule merge-passing algorithm was also proposed to reduce the memory overhead of their algorithm for low to moderate-throughput decoders. Moreover, a parallel soft-input soft-output (SISO) message update mechanism was proposed. Simulation results showed that their algorithm attained a throughput of 1.92 Gbps for a frame length of 2304 bits, power saving of 89.13%, and 69.83% reduction in silicon area with a reduced interconnect length of 60.5%.

Reviriego et al. [10] proposed a majority logic decoder using simple hardware. In this method, during the first iteration of majority logic decoding, a word is detected for errors and suppose if it is without any errors, the remaining iterations get stopped and hence the decoding process is complete. The average decoding time decreases as most of the words of a memory do not possess any errors. This one step majority logic decodable method was also implemented over a similar technique called Euclidean geometry low-density parity-check (EG-LDPC) codes. Their results showed that the method estimated the probability of error detection for various code sizes and different error counts with more accuracy and was proved to be suitable for EG-LDPC codes.

Torrieri et al. [11] proposed a double iterative LDPC based CDMA (code division multiple access) receiver without the use of any training or pilot symbols. An expectation-maximization channel-estimation algorithm for the fading amplitude and interference-plus-noise power spectral density (PSD) is proposed for CDMA systems with low-density parity-check codes. The elimination of pilot symbols simplifies the system design and enhances either information throughput or spectral efficiency by increasing the information-symbol duration. After initial estimates of the fading amplitude and noise PSD are obtained, succeeding values of these parameters are iteratively updated from the channel decoder. These updated estimates are then combined with the received symbols and iteratively passed to the decoder. Experimental results show that this system has higher throughput but bit error rates greater than other systems.

The low-density parity-check codes for turbo multiuser detection in multipath CDMA channel are designed by Wang et al. [12]. They developed a technique for computing the probability density function (pdf) of the output extrinsic messages as a function of the pdf of input extrinsic messages. In case of additive white Gaussian noise channels and asynchronous multipath fading channels, the extrinsic messages can be considered symmetric Gaussian distributed. Simulation results are significant with the computed thresholds and irregular LDPC codes designed outperform the regular ones.

The nonbinary mixed domain LDPC decoders and their finite precision effects were studied by Kim and Sobelman [5]. They used proper scaling techniques and offset-based methods to improve the decoding performance. Besides, a novel Fast Fourier Transform (FFT)-based belief propagation (BP) decoder architecture was proposed to balance the operational load among various processing units. The experimental outcomes demonstrated that their method reduced the number of field programmable gate array slices to 47% compared with other standard FFT-based BP decoders.

Spagnol et al. [4] proposed a modified belief propagation algorithm for Galois Field-GF(2^*m*^) LDPC codes. The algorithm has been implemented with low hardware utilizations in VLSI. Their decoding algorithm used a serial architecture for GF(2^*m*^) are implemented on FPGA. Their results showed that their algorithm was suitable for short to medium length codes.

The serial GF (64)-LDPC decoder proposed by Boutillon et al. [13] was based on Extended Min-Sum algorithm. The algorithm was applied for different code rates and lengths and obtained a performance at less than 0.7 dB from the belief propagation algorithm. Their decoder easily gets adapted for very high Galois Field orders, such as GF(4096) and even higher. Their experimentation results proved that their decoder used less than 20% of decoder area than that of Virtex-4 FPGA at a throughput rate of 2.95 Mbps.

Hwang and Lee [14] studied and discussed various methods used in the construction of Block-Circulant (BC) Reed Solomon based low-density parity-check (RS-LDPC) codes. Their system resulted in a BC form from a random parity-check matrix for RS-LDPC codes. Based on the obtained BC parity-check matrix, switch network and decoder architecture has been built for BC-RS-LDPC codes. The designed BC-RS-LDPC decoder possesses high performance to prove the efficiency of their system. Their experimentation showed that the decoder utilized 1.3 M gates at an operating frequency of 450 MHz with 8 iterations at a throughput rate of 41 Gbps.

The conventional methods in [15–21] stated various design techniques for check node and variable node unit of LDPC decoder. All these techniques focused on error rate reduction and low power consumption by reducing the overhead and area reduction. The proposed methodology stated in this paper is based on Euclidean orthogonal architecture, which determines the severity level of the noises from the received signals in check node unit. If the noise severity on the received data is high (determined by Euclidean orthogonal generator), then it will make the variable node unit unable to decode the received signals. If the noise severity on the received data is high, then it will enable the variable node unit to decode the received signals.

## 3. Architecture of Proposed Decoder

**Figure 1:**
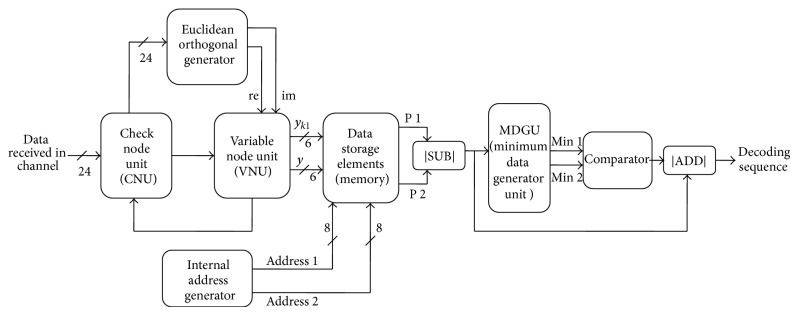
Proposed architecture of decoder.

The proposed method of decoder design comprises several processing elements—variable node unit (VNU), check node unit (CNU), address generator, minimum data generator, and several other modules as shown in Figure 1. The interconnections between CNUs and VNUs are provided by the routing networks. The connectivity provided by the parity-check matrix creates the label for input and output edges of the CNU. After the completion of required number of iterations, the VNU produces the final outputs.

Algorithm of proposed decoding is described in the following.

1. Data with noise is received from the channel and fed into the check node unit of the LDPC decoder.
2. Euclidean orthogonal generator gets the sequence from the check node unit and enable/disable the variable node unit based on the severity level of the noises on the received sequences.
3. The output from variable node unit is stored in the data storage elements which received the address sequence from the address generator module.
4. Minimum data generator unit generates the minimum 1 and minimum 2 data which are then given to the comparator unit.
5. The response from comparator is added with the response from the subtractor unit to produce the decoded signals.

### 3.1. Check Node Unit

**Figure 2:**
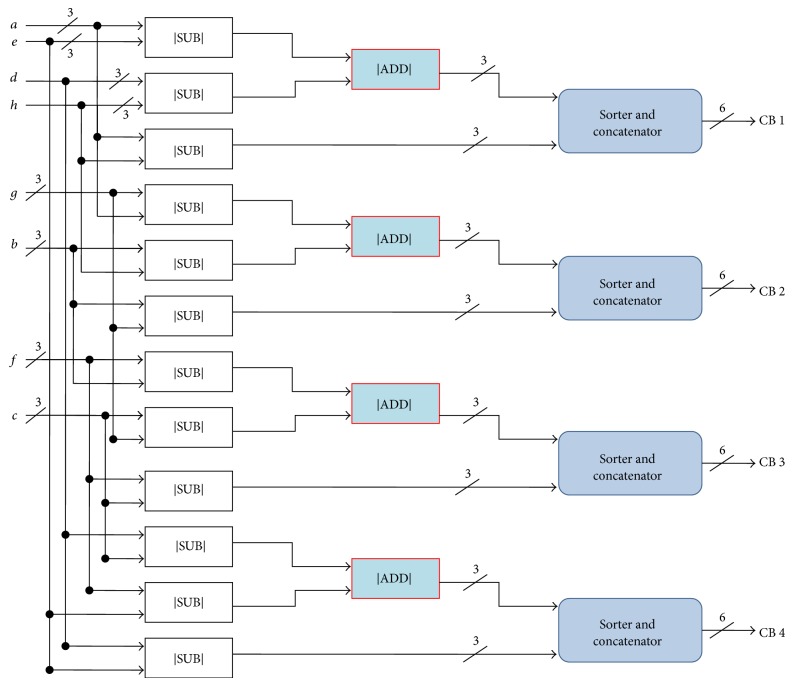
Architecture of check node unit.

The architecture of the check node unit of LDPC decoder is shown in Figure 2. The CNU helps in the determination of the strength of the received signal in the channel. The CNU consists of twelve subtractor (|SUB|) units, four adder (|ADD|) units, and four sorter and concatenator modules which are used in estimating the four directional difference vectors, from which the smallest value of difference is decided. In the proposed design, |ADD| and |SUB| units are required for the design of check node.

The gate count of a single multiplier or division module is much more than the gate count of one adder or subtractor unit; hence only adders and subtractors are fully utilized in our design instead of multipliers and division units, thereby reducing the hardware complexity and cost.

### 3.2. Architecture of Variable Node Unit (VNU)

**Figure 3:**
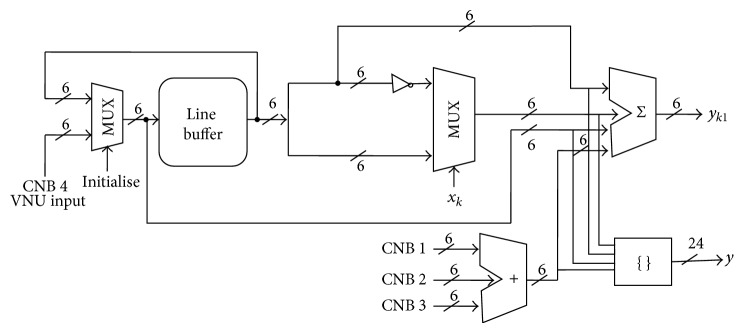
Variable node architecture.

The variable node unit (VNU) as shown in Figure 3 is used in computing the hard decision vector “*x*”. The hard decision vector “*x*” is routed to the check node unit or block (CNB) through the routing network, whose size is smaller than the network size of sum-product algorithm. A single-bit value exists for routing between two nodes. The VNU comprises a flip-detection circuit, line buffer, multiplexed adder, and concatenator. At first, the received signal “*y*” from check node unit passes through a register-feedback assembly to enter the variable node block (VNB). The received values are 6-bit signed-magnitude (SM) values. Let [*sn* : *mn*] be *n*th 4-bit value provided to the *n*th VNB, where *sn* denotes hard decision value and *mn* denotes the magnitude of *sn*. The correlation calculation is made simpler by SM for implementation and the correlator circuit consists of an inverter followed by a 1-bit multiplexer. The block data received from CNU unit have four subblocks, out of which the first three submodule blocks are processed by adder module and last submodule block is processed directly by multiplexer unit.

### 3.3. MDGU

**Figure 4:**
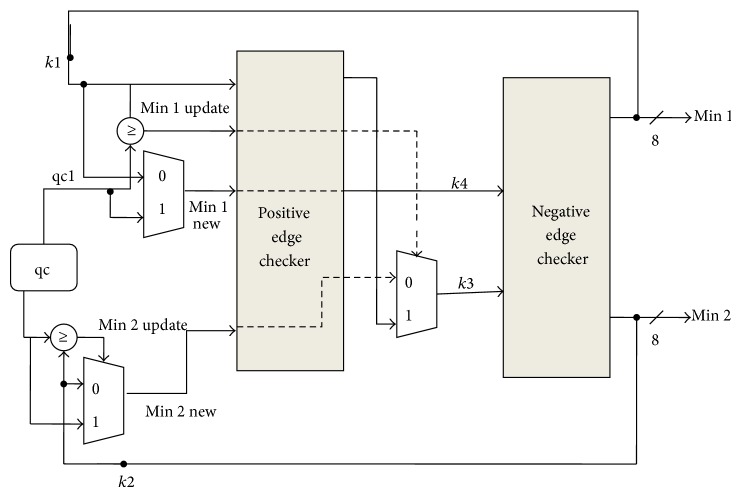
Minimum data generator unit.

A value “qc” is produced at every clock cycle and fed to the minimum data generator unit (MDGU) depicted in Figure 4. Then, “qc” is compared to the updated first and second minimum (min 1 and min 2), that are initialized as the maximum allowed value at the beginning of each check node phase. The minimum of both comparisons (min 1_new and min 2_new) is passed on and sampled on the rising edge of the clock signal, together with the previous first minimum and a flag signaling if min 1 ≠ min 1_new. If min 1_update = 0, then min 2_new is substituted with previous value of min 1. Finally, min 1 and min 2 are updated on the falling edge of the clock, ready, and stable for the next “qc”. Once min 1 and min 2 are produced, they are compared to all the “qc” of the CN, which are delayed by a number of clock cycles equal to the degree of the CN (deg).

### 3.4. Internal Address Generator

**Figure 5:**
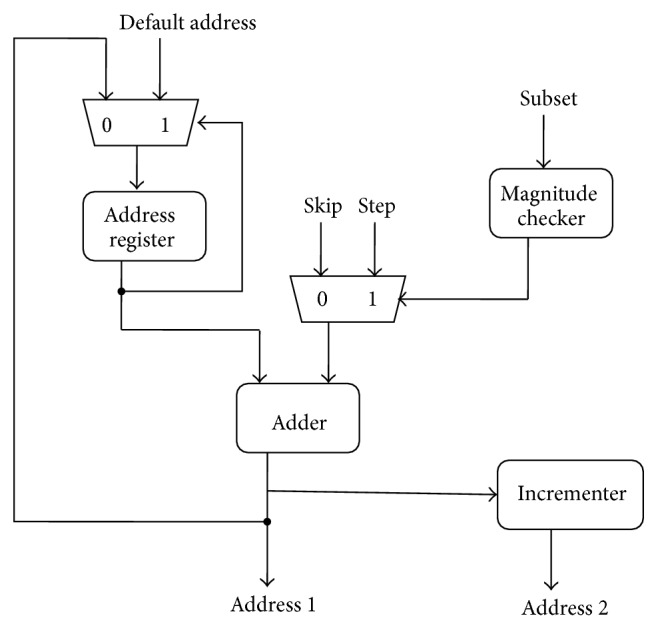
Internal address generator.

Figure 5 shows the block diagram of the internal address generator (IAG) for the input and the output. The instruction memory provides the initial address and SKIP, STEP, and SUBSET fields to address generator. After every cycle is executed, the current address is processed by the IAG for calculating the address of the next set of operands. The output address generator works very similar to that of input address generator, but at the opposite phase of the clock compared to the input address generator. Therefore, the reconfigurable cells (RCs) guarantee the input data to be read in a single clock cycle and write back the output in the opposite phase of the clock. Hence, the RC is ensured to perform one complete computation within a single clock cycle.

### 3.5. Euclidean Orthogonal Generator

**Figure 6:**
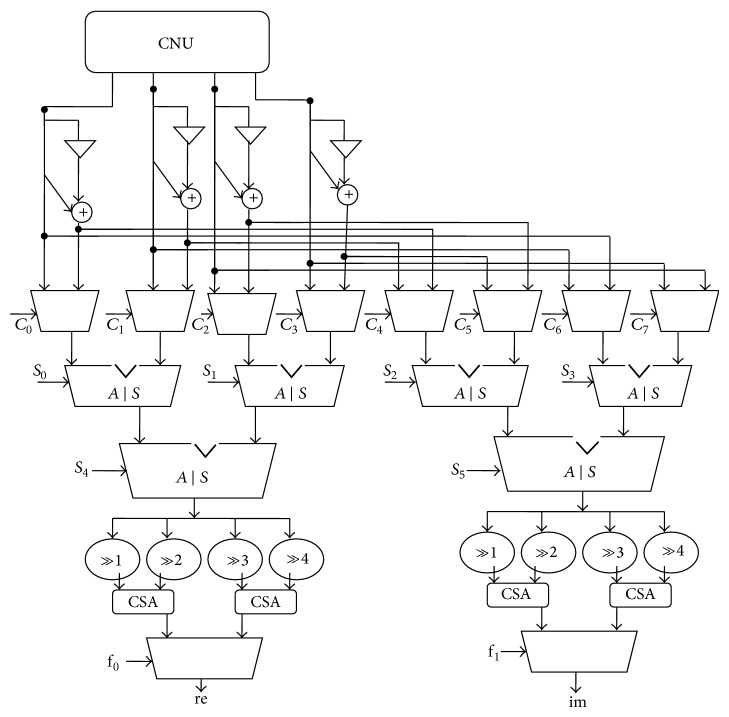
Euclidean orthogonal generator.

It is used to determine the level of noise on the received signal as shown in Figure 6. The output from CNU is fed into the Euclidean orthogonal generator, which consists of adder/subtractor, shifter, and multiplexers. It will produce the real and imaginary part of the received signal from CNU. If the real term is greater than the imaginary term, then the signal from CNU is passed through the VNU in order to decode the signals; else it is discarded due to the high severity of noise on the received sequences.

## 4. Results and Discussion

The architecture of LDPC decoder designed in this work is more suitable for low power applications and is verified using Xilinx 9.2i and Modelsim 5.8 on several Virtex family devices. The proposed decoder is simulated on various FPGA devices for its performance analysis. The input for proposed decoder is taken from the real time mobile environment with noises. The clock frequency for the simulation is 200 MHz with 50-50 on-off clock period as initial simulation setup. The designed decoder made use of 100 LUTs and 51 slices with an operating frequency of 200 MHz at the maximum. The results show that this proposed decoder consumes very low power in terms of Look Up Tables, FPGA slices, and gate counts. The power and current consumptions are verified for different devices of the Virtex family and tabulated in Table 1. The performances of the proposed decoder have been analyzed in terms of number of Look Up Tables (LUTs), number of Slices (S), gate counts (GC), Current Consumption (CC), and Power Consumption (PC).  Table 2 explains the performance evaluation of the decoder in terms of hardware used and Table 3 compares the decoding latency of other decoding methods with proposed decoder.

**Figure 7:**
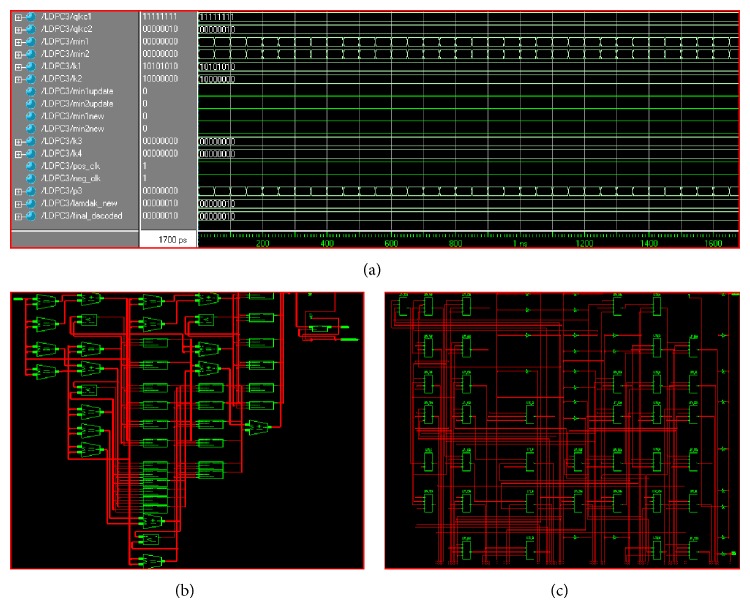
(a) simulation screenshot indicating decoded sequence, (b) RTL schematic of proposed design, and (c) technology schematic of proposed design.

The operation of our proposed decoder is illustrated in Figure 7, which shows the RTL and technology schematic views of the proposed technique are presented.

We have simulated the proposed code and then the proposed LDPC code is downloaded in to the FPGA device for its performance measurement. The power consumption of the proposed LDPC architecture is measured using xprimer or power analyzer tool available in the Xilinx tool. The input to the FPGA unit is given from the real time mobile signal received from the channel with noises. The LDPC decoder proposed in this paper has been compared in terms of power utilizations by evaluating it over different Virtex devices. The LDPC decoder in this work was mainly designed for the purpose of reducing the power levels during operation. Table 1 already discussed the power utilizations of different Virtex devices and it has been shown that power consumptions of such devices are at lower levels.

Several performance assessment parameters and resource utilization parameters were determined for the proposed LDPC decoder. The current study mainly focuses on low power applications and hence the pipelined LDPC decoder is being designed and developed for this purpose. The proposed LDPC decoding architecture is synthesized using Xilinx project navigator version 9.2i tool. The decoding latency obtained for both check node and variable node unit implementations in the proposed architecture is about 28 ns, whereas the decoding latencies obtained by Kim and Sobelman [5] and Spagnol et al. [4] were about 56.36 ns and 50.05 ns, respectively, thereby proving that this proposed LDPC decoder design performs better than conventional decoding methods.

The proposed architecture consumed 4-input LUTs of about 100, which provides a low hardware complexity compared to Kim and Sobelman [5] and Spagnol et al. [4], which consumed 6422 LUTs and 12924 LUTs, respectively. The power consumptions of various methodologies are compared in Table 4.

## 5. Conclusion

We have proposed the design of high efficient LDPC decoder architecture for implementations in low power applications. The design of Euclidean orthogonal generator has been proposed in this paper, which is a part of LDPC decoder architecture. The Euclidean orthogonal generator proposed in this work reduces the error rate of the proposed LDPC architecture. The design discussed produced better results than other conventional decoder architectures with lesser hardware consumption and power utilization of about 27 mW, which is more suitable for most of the low power applications.

## Figures and Tables

**Table 1:** Performance analysis of power and current consumptions of Virtex processors.

Family	Device specifications	Power consumption (mW)	Current consumption (mA)
Virtex-E	XCV50E	151	82
Virtex5	XC5VLX30	267	208
Virtex4	XC4VLX15	253	127
Virtex	XCV50	27	10

**Table 2:** Performance evaluation of hardware utilizaions.

Hardware utilization parameters	Hardware consumption
Slices	51
LUTs	100
Gate counts	3330

**Table 3:** Analysis of decoding latency.

Methodology	Decoding latency (ns)	CMOS technology	Frequency (MHz)
Proposed	22.02	45 nm	200
Spagnol et al. (2009) [4]	50.05	65 nm	300
Kim and Sobelman (2013) [5]	56.36	180 nm	450

**Table 4:** Comparison of power consumptions of conventional methods.

Methodology	Power consumption (mW)^**^	CMOS technology	Energy consumption (nJ)^*^
Proposed method	27	45 nm	104.7
Ismail et al. (2013) [6]	33.14	65 nm	144.9
Blanksby and Howland (2002) [7]	690	90 nm	256.1
Mansour and Shanbhag (2006) [8]	787	65 nm	320.0
Mohsenin et al. (2010) [9]	1359	180 nm	389.9

^*^Energy consumption of one clock period. This comparison is only for the energy in one clock cycle, which maps to the power consumption.

^**^Code rate is 0.96 and word length is 7 bits with 8 iterations.
